# Phytochrome B and REVEILLE1/2-mediated signalling controls seed dormancy and germination in *Arabidopsis*

**DOI:** 10.1038/ncomms12377

**Published:** 2016-08-10

**Authors:** Zhimin Jiang, Gang Xu, Yanjun Jing, Weijiang Tang, Rongcheng Lin

**Affiliations:** 1 Key Laboratory of Photobiology, Institute of Botany, Chinese Academy of Sciences, Nanxincun 20, Xiangshan, Beijing 100093, China; 2 University of the Chinese Academy of Sciences, Beijing 100049, China; 3 CAS Center for Excellence in Molecular Plant Sciences, Chinese Academy of Sciences, Beijing 100093, China

## Abstract

Seeds maintain a dormant state to withstand adverse conditions and germinate when conditions become favourable to give rise to a new generation of flowering plants. Seed dormancy and germination are tightly controlled by internal and external signals. Although phytochrome photoreceptors are proposed to regulate primary seed dormancy, the underlying molecular mechanism remains elusive. Here we show that the REVEILLE1 (RVE1) and RVE2 transcription factors promote primary seed dormancy and repress red/far-red-light-reversible germination downstream of phytochrome B (phyB) in *Arabidopsis thaliana*. *RVE1* and *RVE2* expression is downregulated after imbibition and by phyB. RVE1 directly binds to the promoter of *GIBBERELLIN 3-OXIDASE 2*, inhibits its transcription and thus suppresses the biosynthesis of bioactive gibberellins. In addition, DELAY OF GERMINATION 1 also acts downstream of phyB. This study identifies a signalling pathway that integrates environmental light input with internal factors to control both seed dormancy and germination.

## Abstract (web summary)

Seed dormancy prevents germination under adverse environmental conditions. Here the authors show that the RVE1 and RVE2 transcription factors suppress biosynthesis of the germination-promoting hormone GA and act downstream of phytochrome signalling to promote dormancy and repress light-induced germination.

## Graphical abstract

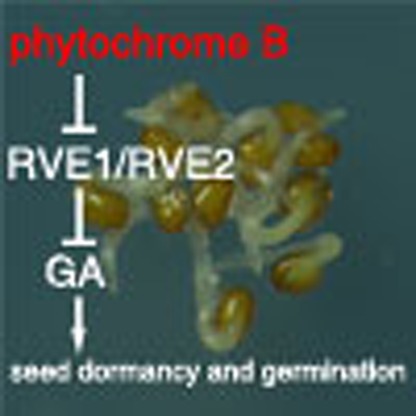

Seeds mediate the alternation of generations in flowering plants and have been a staple food throughout human civilization. Primary seed dormancy is acquired during seed maturation and reaches a high level in freshly harvested seeds and maintained for a certain period that allows seeds to survive under unfavourable conditions and prevents pre-harvest sprouting, and is thus an important aspect of plant fitness^1^,^2^,^3^. Under optimal conditions, the release of dormancy by after-ripening and the successful germination and establishment of a robust seedling are critical for the propagation of the plant species^4^,^5^. Dormancy and germination are two distinct but closely connected physiological processes. The dormancy-to-germination transition is a critical developmental step in the life cycle of plants that is determined by both genetic factors and environmental influences^2^,^3^,^4^. The phytohormones gibberellin (GA) and abscisic acid (ABA) primarily and antagonistically regulate the seed status; GA represses dormancy and promotes germination, whereas ABA has the opposite effects. The signalling pathways that GA and ABA control seed germination have been extensively studied^2^,^6^,^7^,^8^,^9^.

Previous genetic analyses have revealed many regulators that affect the induction, maintenance and release of seed dormancy^2^,^3^. Studies of natural variation have identified multiple quantitative trait loci (QTL) that contribute to dormancy in wild populations of *Arabidopsis thaliana* and some crops^10^,^11^,^12^,^13^,^14^,^15^. Among them, *DELAY OF GERMINATION 1* (*DOG1*) is a major QTL in a recombinant inbred line population of *Arabidopsis*^10^,^16^. DOG1 protein levels in freshly harvested dry seeds strongly correlate with the time required for after-ripening^17^. A recent study showed that DOG1 regulates primary seed dormancy through a microRNA pathway^18^. However, the molecular function and regulation of *DOG1* remain elusive.

Light is a major environmental signal that oppositely modulates the levels of GA and ABA, and affects seed germination^8^,^19^,^20^. Among plant photoreceptors, the red and far-red-light-absorbing phytochromes are essential for light promotion of germination^21^. In the model species *Arabidopsis*, five genes (*PHYA* to *PHYE*) encode phytochrome apoproteins^22^,^23^. Phytochrome B (phyB) predominantly triggers red/far-red-light-reversible seed germination, whereas phyA mediates distinct, very low fluence responses in red and far-red light^24^,^25^,^26^,^27^,^28^,^29^,^30^,^31^. phyA- and phyB-dependent induction of germination are spatially separated in the endosperm and embryo^32^. phyE is required for germination in continuous far-red light^33^. A recent study shows that phyE and phyD stimulate germination at very low red/far-red ratios and, surprisingly, phyC antagonizes the promotion of germination by light^34^. At the molecular level, far-red light converts phyochromes into the inactive Pr form, which inhibits seed germination, whereas a subsequent red-light pulse reverts it to active Pfr and induces germination^22^,^35^. Light-activated phyB interacts with and promotes the degradation of a negative regulator, PHYTOCHROME-INTERACTING FACTOR 1 (PIF1, also known as PIL5)^36^,^37^. PIF1 directly regulates the expression of several downstream genes, including *GA-INSENSITIVE*, *REPRESSOR OF GA1-3* and *SOMNUS*, which modulates GA responsiveness, GA and ABA biosynthesis and subsequent seed germination^38^,^39^,^40^,^41^. PIF6 was previously shown to regulate the primary seed dormancy^42^. Although phytochromes are involved in regulating seed dormancy^21^,^28^,^43^, the underlying molecular mechanism was hitherto unknown.

REVEILLE1 (RVE1) belongs to a subfamily of Myb-like transcription factors that includes CIRCADIAN CLOCK-ASSOCIATED 1 (CCA1) and LATE ELONGATED HYPOCOTYL clock components^44^,^45^. RVE1 regulates hypocotyl growth by integrating the circadian clock and auxin pathways^44^. We previously revealed that RVE1 modulates chlorophyll biosynthesis and seedling de-etiolation in *Arabidopsis*^46^. In this study, we identified RVE1 and RVE2 as activators of seed dormancy and further provided molecular and genetic evidence to show that RVE1 and RVE2 promote primary seed dormancy and repress red/far-red-light-mediated germination downstream of phyB in *A. thaliana*. We found that the transcription of *RVE1*, *RVE2* and *DOG1* is reduced by phyB-Pfr, the active form of phyB. We also demonstrate that RVE1 directly inhibits *GIBBERELLIN 3-OXIDASE 2* (*GA3ox2*) transcription and subsequently suppresses bioactive GA biosynthesis. Therefore, we reveal a genetic pathway that links light input with internal factors to control seed dormancy and germination that can potentially optimize seed adaptability to changing environments.

## Results

### RVE1 and RVE2 regulate seed dormancy and germination

**Figure 1:**
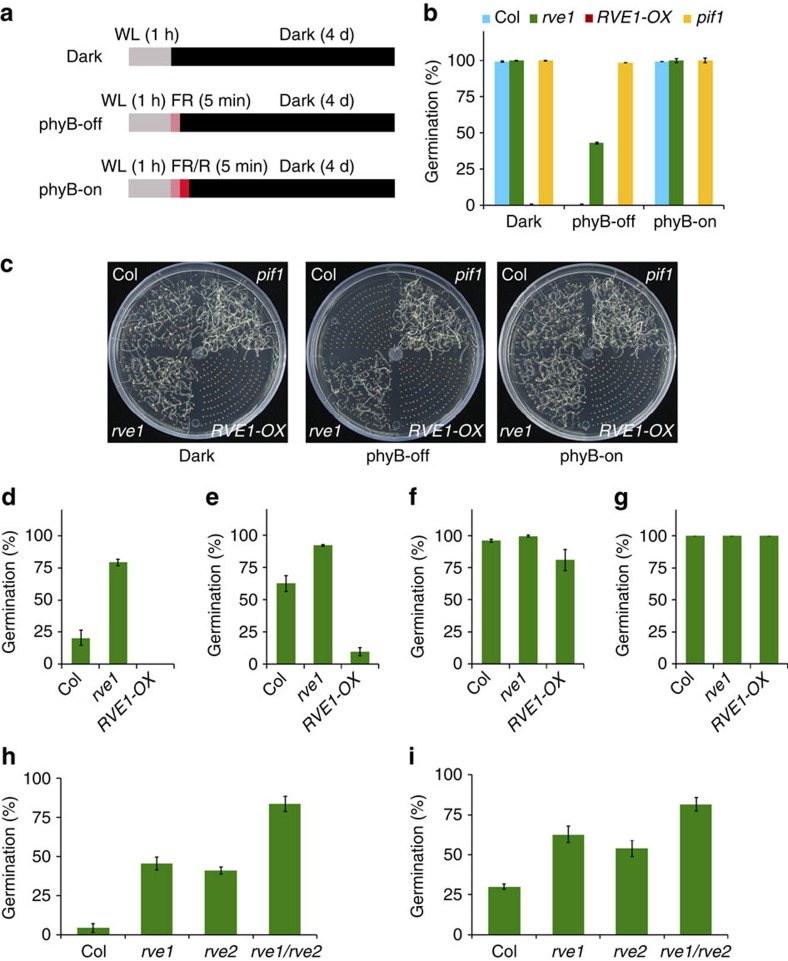
RVE1 regulates both seed dormancy and germination. (**a**) Light irradiation treatment in the experiments. Post-harvest seeds were irradiated with white light (WL) for 1 h (starting from seed sterilization) and were then exposed to far-red (FR) light for 5 min (phyB-off) or followed by 5 min of red (R) light (phyB-on). Seeds were then kept in darkness and germination frequencies were recorded after 4 days. (**b**) Quantification of the germination frequencies of seeds under different conditions as shown in **a**. (**c**) Representative images of seed germination assays of Col, *rve1* and *RVE1-OX* seeds under the light conditions shown in **a**. (**d**–**g**) Percentage of seed germination. Freshly harvested seeds were kept in darkness (**d**) or under white light (**e**) for 3 days, or seeds were stratified at 4 °C for 1 day (**f**) or 3 days (**g**) in darkness before being exposed to 3 days of white light treatment at 22 °C. (**h**) Germination percentage of post-harvest seeds of *rve1*, *rve2* and *rve1/rve2* grown under the phyB-off condition. (**i**) Dormancy phenotype of freshly harvested seeds of *rve1*, *rve2* and *rve1/rve2* grown in darkness for 4 days. For **b** and **d**–**i**, mean±s.d., *n*=3.

We first examined the role of RVE1 in light-induced seed germination using 2- to 5-month post-harvest seeds. The after-ripening seeds were exposed to different light conditions without cold stratification (Fig. 1a). The phyB photoreceptor positively controls red/far-red reversible seed germination^25^,^26^,^27^. As shown in Fig. 1b,c, under phyB-off conditions (grown in darkness interrupted by a 5-min pulse of far-red light to inactivate phyB), Columbia (Col) wild-type seeds did not germinate, whereas the germination frequency of *rve1*-null mutant (Supplementary Fig. 1a,b) was close to 40%. Under darkness or phyB-on conditions (grown in darkness interrupted by a 5-min pulse of far-red light followed by 5-min pulse of red light to activate phyB), 100% of the *rve1* seeds germinated, similar to Col. Remarkably, *RVE1* overexpression (*RVE1-OX*) transgenic seeds failed to germinate under all conditions (Fig. 1b,c). It was further supported by a time-course germination assay (Supplementary Fig. 1c). The *pif1* seeds, which were used as a control, germinated under all conditions tested, as reported previously (Fig. 1b,c)^38^. These observations suggest that RVE1 negatively regulates phyB-mediated seed germination.

Next, we asked whether RVE1 is involved in seed dormancy using freshly harvested seeds. We found that ∼80% of the *rve1* seeds germinated compared with ∼20% of Col seeds, whereas *RVE1-OX* seeds failed to germinate in dark condition (Fig. 1d). Under white light conditions, ∼60% of Col seeds germinated, whereas ∼90% of *rve1* seeds and <10% of *RVE1-OX* seeds germinated (Fig. 1e). Notably, after 1 day of cold stratification followed by 3 days of exposure to white light treatment at 22 °C, almost all Col seeds germinated, whereas ∼80% of *RVE1-OX* seeds germinated (Fig. 1f). This reduced germination rate was not due to developmental defects, because all of the *RVE1-OX* seeds germinated after 3 days of cold stratification (Fig. 1g). In addition, a far-red light pulse (phyB-off) inhibited germination of *rve1*, whereas a further red light pulse induced germination of freshly harvested seeds (Supplementary Fig. 1d). Another line of *RVE1* overexpression plant (*35S:Myc-RVE1*) showed a similar response to *RVE1-OX* (Supplementary Fig. 1e).

*RVE2* is the closest homolog of *RVE1* (ref. 47). Similar to *rve1*, *rve2*-null mutant seeds displayed increased seed germination (Supplementary Fig. 2) and the *rve1/rve2* double mutant had even higher germination frequencies than the single mutants (Fig. 1h,i). Overexpression of *RVE2* (*RVE2-OX*) resulted in reduced phyB-mediated seed germination (Supplementary Fig. 2c). *RVE2-OX* also conferred the dormancy response (Supplementary Fig. 2d). Taken together, these phenotypic analyses indicate that RVE1 and RVE2 play additive and dual roles in controlling both primary seed dormancy and phyB-dependent seed germination.

### RVE1 and DOG1 act genetically downstream of phyB

**Figure 2:**
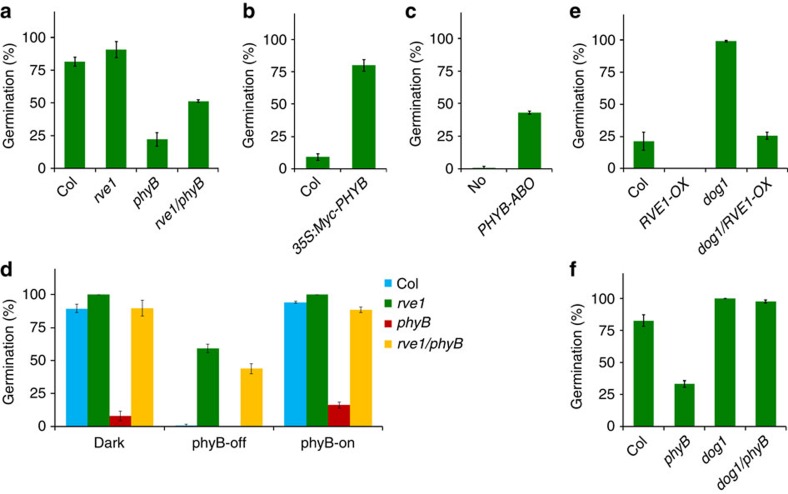
Genetic interaction with phyB. (**a**) Germination frequency of freshly harvested seeds of Col, *phyB-9*, *rve1* and *rve1/phyB* incubated under white light for 4 days. (**b**) Germination frequency of freshly harvested Col wild-type and *35S:Myc-PHYB* transgenic seeds incubated in darkness for 4 days. (**c**) Germination frequency of freshly harvested Nossen (No)-0 wild-type and *PHYB-ABO* transgenic seeds incubated in darkness for 4 days. (**d**) Germination frequency of post-harvest seeds of Col, *phyB-9*, *rve1* and *rve1/phyB* incubated under different light conditions as indicated in Fig. 1a. (**e**) Seed dormancy response of freshly harvested seeds of Col, *RVE1-OX*, *dog1* and *dog1*/*RVE1-OX* grown in darkness for 4 days. (**f**) Dormancy response of freshly harvested seeds of Col, *phyB*, *dog1* and *dog1/phyB* incubated under white light for 4 days. For **a**–**f**, mean±s.d., *n*=3.

Early studies suggest the involvement of phyB in seed germination of freshly harvested seeds^21^,^28^. We further observed that freshly harvested *phyB* mutant seeds exhibited a strong dormant phenotype under white light (Fig. 2a), whereas two lines of *PHYB* overexpression transgenic seeds, *35S:Myc-PHYB* (Supplementary Fig. 3a) and *PHYB-ABO*, had a greater germination frequency than their corresponding wild-type controls in darkness (Fig. 2b,c). Interestingly, *PHYB* overexpression seeds had 100% germination frequencies compared with 30–35% for wild-type seeds under complete darkness (Supplementary Fig. 3b). These data confirm that phyB suppresses primary seed dormancy. We then generated a *rve1/phyB* double mutant by genetic crossing between *rve1* and *phyB-9*. The reduced dormancy of *rve1* was partly suppressed by *phyB* mutation in the *rve1/phyB* double mutant (Fig. 2a). Moreover, the post-harvest *phyB* seeds had low germination rates in darkness (Fig. 2d). Notably, homozygous *rve1/phyB* had germination frequencies close to those of *rve1* under all conditions (Fig. 2d). These results suggest that RVE1 acts genetically downstream of phyB to regulate primary seed dormancy and red/far-red light reversible germination.

DOG1 is an important suppressor of seed dormancy^16^,^17^,^18^. To examine the relationship between RVE1 and DOG1, we introduced *RVE1-OX* into the *dog1*-null mutant background. Overexpression of *RVE1* complemented the non-dormant phenotype of *dog1* in the *dog1/RVE1-OX* homozygous seeds in darkness (Fig. 2e). Furthermore, DOG1 acted downstream of phyB in the regulation of primary seed dormancy (Fig. 2f).

### *RVE1/2* and *DOG1* expression is inhibited by phyB

**Figure 3:**
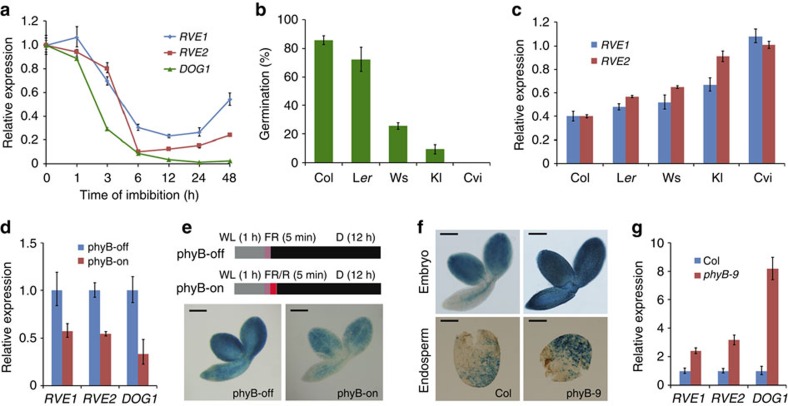
Expression pattern of *RVE1* and *RVE2*. (**a**) Relative expression of *RVE1*, *RVE2* and *DOG1* during imbibition. Freshly harvested Col seeds were imbibed in 0.6% agar plates for the indicated time. (**b**) Germination frequency of freshly harvested seeds from Col, Landsberg *erecta* (L*er*), Wassilewskija (Ws), Köln (Kl) and Cape Verde Islands (Cvi) ecotypes. Seeds were grown under white light conditions for 4 days. (**c**) Relative expression levels of *RVE1* and *RVE2* in different ecotypes. Total RNA was isolated from seeds imbibed under light for 12 h. (**d**) Quantitative reverse transcriptase–PCR of *RVE1* and *RVE2* in post-harvest Col seeds grown under phyB-off and phyB-on conditions. (**e**) GUS staining of *RVE1p:GUS* transgenic seeds after phyB-off and phyB-on treatments as indicated in the top panels. D, dark; FR, far-red light; R, red light; WL, white light. (**f**) GUS staining of freshly harvested Col and *phyB* seeds harbouring *RVE1p:GUS.* The seeds were incubated in darkness for 12 h. Scale bars, 200 μm (**e**,**f**). (**g**) Relative expression of *RVE1*, *RVE2* and *DOG1* in Col and the *phyB-9* mutant. Freshly harvested seeds were imbibed for 36 h in darkness. For **a**–**d** and **g**, mean±s.d., *n*=3.

Next, we evaluated the expression pattern of *RVE1*, *RVE2* and *DOG1* during embryogenesis from public data repositories^48^. The transcript levels of *RVE1*, *RVE2* and *DOG1* increase gradually during seed development (Supplementary Fig. 4 and ref. 17), indicating that they are required for dormancy induction. However, the other *RVE* genes and *PIF1* did not display such an expression mode (Supplementary Fig. 4). Quantitative reverse transcriptase–PCR showed that, similar to *DOG1*, *RVE1* and *RVE2* expression was rapidly decreased in freshly harvested wild-type seeds during imbibition (Fig. 3a)^17^. We next examined whether the expression of *RVE1* and *RVE2* correlates with the level of dormancy in different ecotypes. Under white light, freshly harvested seeds of Col and Landsberg *erecta* accessions were non-dormant and those of Wassilewskija and Köln ecotypes were partially dormant, whereas Cape Verde Island seeds were completely dormant (Fig. 3b). Conversely, the level of *RVE1* and *RVE2* transcripts increased with the degree of dormancy of these ecotypes (Fig. 3c). Furthermore, the expression of *RVE1*, *RVE2* and *DOG1* was drastically reduced by a red light pulse (phyB-on) in Col seeds (Fig. 3d). We then generated *RVE1p:GUS* transgenic plants in which β-glucuronidase (GUS) reporter expression was driven by the *RVE1* promoter sequence (1.4 kb upstream of the ATG start codon). Under phyB-off condition, the cotyledons and radical of *RVE1p:GUS* seeds were stained in blue, whereas staining was greatly reduced under phyB-on conditions (Fig. 3e). *RVE1p:GUS* was also introduced into the *phyB* mutant. *GUS* expression in both the embryo and endosperm of fresh seeds was remarkably increased in *phyB* compared with in Col control (Fig. 3f). Consistently, the transcript levels of *RVE1*, *RVE2* and *DOG1* were increased in *phyB* (Fig. 3g). However, a chromatin co-immunoprecipitation (ChIP) assay showed that phyB was not recruited to the chromatin of *RVE1*, *RVE2* and *DOG1* genes (Supplementary Fig. 5). These experiments confirm that *RVE1*, *RVE2* and *DOG1* expression are repressed after imbibition and by active form of phyB.

### RVE1 directly and specifically represses *GA3ox2* expression

**Figure 4:**
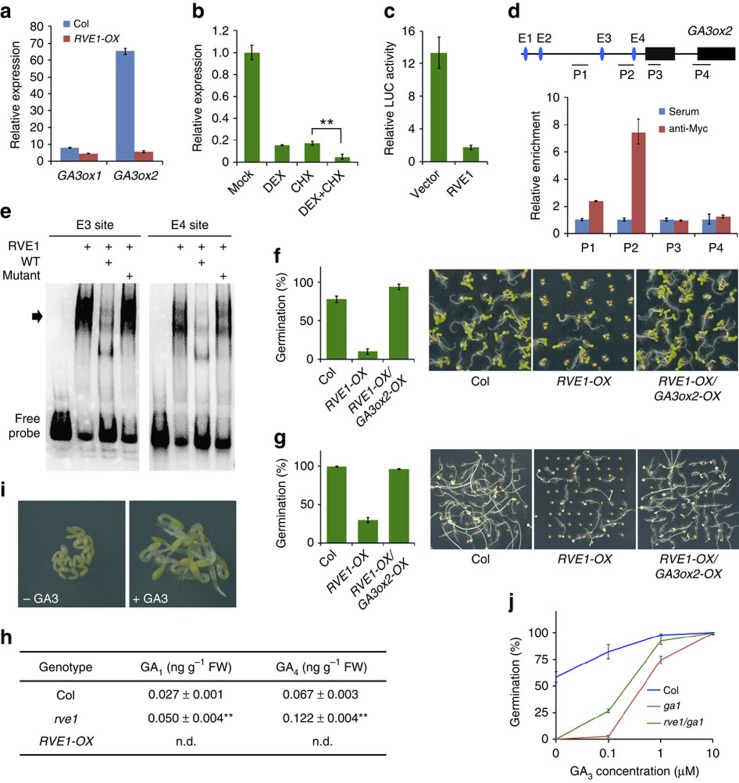
RVE1 regulates GA biosynthesis by directly modulating *GA3ox2* expression. (**a**) Relative expression levels of *GA3ox1* and *GA3ox2* in Col and *RVE1-OX* grown under phyB-on conditions. (**b**) Relative expression levels of *GA3ox2* in *35S:RVE1-GR* transgenic seeds incubated in darkness for 24 h without (Mock, 0.05% dimethy; sulfoxide (DMSO)) or with 5 μM DEX and/or 50 μM cycloheximide (CHX). Asterisks denote statistically significant difference (*P*<0.01, Student's *t*-test). (**c**) Transient transcriptional assay of *GA3ox2p:LUC* by *RVE1* overexpression or empty vector control in *Arabidopsis* protoplasts. (**d**) ChIP assay of *GA3ox2* DNA precipitated in *35S:Myc-RVE1* by Myc antibody or serum control. Post-harvested *35S:Myc-RVE1* seeds were incubated under phyB-on condition. The top diagram indicates the genomic structure of *GA3ox2*. Black boxes indicate exons. P1 to P4 fragments show PCR regions in the ChIP assay and E1 to E4 sites are putative EE-expand motifs. (**e**) EMSA assay. The oligos were synthesized by fusing three copies of the E3 or E4 motifs and their flanking sequences. The oligos labelled with biotin were used for probes and incubated with or without RVE1 recombinant proteins. Excess amounts (× 50) of unlabelled wild-type (WT) or mutant oligos were added as the competitors. Arrow denotes shifted bands of protein–DNA complexes. (**f**) Seed dormancy phenotype of Col, *RVE1-OX* and *RVE1-OX*/*GA3ox2-OX* transgenic seeds grown under white light for 4 days. (**g**) Light-mediated seed germination phenotype of Col, *RVE1-OX* and *RVE1-OX*/*GA3ox2-OX* transgenic seeds grown under phyB-on conditions. (**h**) Measurement of GA_1_ and GA_4_ contents of freshly harvested Col, *rve1* and *RVE1-OX* seeds imbibed in darkness for 24 h. Data are means±s.d., *n*=3. n.d., not detected. Asterisks indicate significant differences from Col using Student's *t*-test (*P*<0.01). (**i**) Germination phenotype of *RVE1-OX* in the absence or presence of exogenous GA_3_ (10 μM). Seed coats were removed to facilitate GA_3_ absorption and embryos were incubated in darkness for 2 days. (**j**) Germination percentage of Col, *ga1* and *rve1/ga1* in the absence or presence of various concentrations of exogenous GA_3_. Freshly harvested seeds were incubated under white light for 3 days. For all column figures, mean±s.d., *n*=3.

Seed dormancy and germination are predominantly regulated by the balance of GA and ABA levels^2^,^7^,^8^. To reveal the molecular mechanism underlying RVE1 function, we examined the expression of most metabolic genes of the GA and ABA biosynthetic pathways between Col and *RVE1-OX* seeds grown under phyB-on conditions. The GA biosynthetic pathway is catalysed by GA 20-oxidase and GA 3-oxidase (GA3ox) to produce bioactive hormones, which are subsequently deactivated by multiple classes of enzymes such as GA 2-oxidase^49^. ABA is a sesquiterpenoid derived from carotenoids by a series of enzymes^50^. The expression levels of several ABA metabolic genes, including *ABA-DEFICIENT 1* (*ABA1*), *ABA3*, *NINE-CIS-EPOXYCAROTENOID DEOXYGENASE 5* and *CYTOCHROME P450 FAMILY 707 SUBFAMILY A POLYPEPTIDE 2* in *RVE1-OX* were about 1.5- to 2-fold of those in Col, whereas the transcript levels of *ABA4*, *CYP707A3* and two GA metabolic genes, *GA20ox1* and *GA3ox1*, were decreased in *RVE1-OX* by 1- to 2-fold as compared with Col (Fig. 4a and Supplementary Fig. 6). Most remarkably, *GA3ox2* expression was drastically decreased by ∼13-fold in *RVE1-OX* seeds (Fig. 4a). These analyses suggest that RVE1 regulates the expression of both ABA and GA biosynthetic genes.

To test whether RVE1 directly regulates the expression of these genes, we generated *35S:RVE1-GR* transgenic lines in which *RVE1* is fused to a sequence encoding the steroid-binding domain of the rat glucocorticoid receptor (GR) to control its nuclear translocation. In the presence of the glucocorticoid hormone dexamethasone (DEX), *35S:RVE1-GR* showed the long hypocotyl phenotype, as did *RVE1-OX* (Supplementary Fig. 7a). With or without cycloheximide, a reagent that blocks protein synthesis, DEX treatment caused the transcript level of *GA3ox2*, but not of the other GA and ABA metabolic genes tested, to drastically decrease (Fig. 4b and Supplementary Fig. 7b), suggesting that repression of *GA3ox2* requires nuclear targeting of RVE1. Next, we carried out a transient expression assay in *Arabidopsis* mesophyll protoplasts and found that RVE1 greatly repressed the expression of the luciferase (LUC) reporter gene driven by the *GA3ox2* promoter (Fig. 4c). In agreement with this observation, RVE1 possesses transcriptional repression activity (Supplementary Fig. 8).

To confirm the direct effect of RVE1 on *GA3ox2* transcription, we performed ChIP assay using *35S:Myc-RVE1* seeds and Myc antibody. In contrast to the serum control, the Myc antibody pulled down the P2 fragment and, to a less extent, of P1 fragment in the promoter region of *GA3ox2*, but not of *GA3ox1*, in samples prepared under phyB-on conditions (Fig. 4d and Supplementary Fig. 9). CCA1 is occupied at DNA regions containing several conserved motifs, such as evening element (EE) and EE-expand sequences^51^. As RVE1 and CCA1 are in the same subfamily of Myb-like proteins^44^, we reasoned that RVE1 might also bind to target genes via these motifs. By analysing the promoter sequence of *GA3ox2*, we found that there are four putative EE-expand motifs, sites E1 (5′-AGATATGA-3′) and E2 (5′-GGATATGT-3′) being upstream of P1 fragment, site E3 (5′-GATATTA-3′) locating between P1 and P2 fragment, and site E4 (5′-TCATATCA-3′) being adjacent to P2 fragment (Fig. 4d). E3 and E4 were most likely to be the binding sites of RVE1 based on the ChIP result. To test this possibility, we purified RVE1 recombinant protein and carried out an electrophoresis mobility shift assay (EMSA). As shown in Fig. 4e, incubation with RVE1 protein caused strong mobility shift bands of probes for both E3 and E4 sites. Most intriguingly, addition of excess unlabelled wild-type oligos significantly reduced the binding, whereas addition of mutant oligos (5′-GATATTA-3′ changed to 5′-gggATag-3′ in E3, and 5′-TCATATCA-3′ changed to 5′-cgtctcag-3′ in E4) did not affect the shift (Fig. 4e), indicating that RVE1 directly binds to the specific EE-expands motifs of *GA3ox2 in vitro*. These results together confirm that RVE1 directly represses *GA3ox2* expression.

### RVE1 regulates GA biosynthesis

Next, we constructed transgenic plants overexpressing *GA3ox2* in the *RVE1-OX* background (*RVE1-OX/GA3ox2-OX*) and used the resulting homozygous seeds to examine the genetic relationship between *GA3ox2* and *RVE1*. Overexpression of *GA3ox2* complemented both the dormancy and germination phenotypes of *RVE1-OX* in *RVE1-OX/GA3ox2-OX* (Fig. 4f,g), suggesting that *GA3ox2* acts genetically downstream of *RVE1*. *GA3ox2* encodes a GA3ox, which catalyses the production of bioactive GAs^49^. We reasoned that the impaired expression of *GA3ox2* in the *RVE1* mutant or overexpression seeds would alter endogenous GA levels. As expected, the content of bioactive forms of GA, including GA_1_ and GA_4_, nearly doubled in freshly harvested *rve1* seeds, but were undetectable in *RVE1-OX* seeds, compared with the Col control (Fig. 4h). However, ABA levels in *rve1* and *rve1/rve2* were indistinguishable from those in the wild-type control (Supplementary Fig. 10).

We then asked whether exogenous application of GA_3_ could rescue the *RVE1-OX* phenotype. As shown in Fig. 4i, GA_3_ treatment indeed rescued germination of *RVE1-OX* seeds. GA biosynthetic mutants such as *gibberellic acid-requiring1-3*, lacking synthesis of *de novo* GA due to a defect in an early step of the GA metabolic pathway, do not germinate even under favourable conditions^52^. The *rve1/ga1* double mutant was unable to germinate, as was the *ga1* single mutant, but germinated better than *ga1* in the presence of exogenous GA_3_ (Fig. 4j), suggesting that RVE1 function requires GA biosynthesis. Taken together, these data indicate that RVE1 indeed specifically controls GA biosynthesis through direct regulation of *GA3ox2* and probably also of other genes in an indirect manner.

## Discussion

Phytochrome-mediated red/far-red-light-reversible regulation on seed germination has been extensively studied and signalling pathways have been proposed^22^,^41^,^53^. The involvement of light in regulating primary seed dormancy was previously observed in phytochrome-deficient mutants^21^,^28^,^54^; however, the underlying molecular mechanism is not understood. In this report, we used freshly harvested seeds to study primary dormancy and after-ripened seeds to determine germination, and identified RVE1 and RVE2 as two novel factors in regulating both processes by light. We show that RVE1 and RVE2 transcription factors function additively downstream of phyB in controlling primary seed dormancy and germination (Figs 1 and 2), supporting the idea that dormancy is regulated by phytochromes^21^,^42^. Expression of *RVE1*, *RVE2* and *DOG1* is increased during seed development probably to induce seed dormancy (Supplementary Fig. 4)^17^. phyB apoprotein accumulates during seed maturation^25^ and is activated after imbibition to repress *RVE1*, *RVE2* and *DOG1* expression (Fig. 3). Overexpressing *PHYB* breaks dormancy even under darkness, possibly due to seeds producing Pfr form of phyB in the maternal environment during maturation and its constitutive inhibition of the transcription of *RVE1/2* and *DOG1*. As phytochromes relay light signals to PIF proteins^22^,^35^ and phyB is not able to associate with the DNA sequences of *RVE1*, *RVE2* and *DOG1* (Supplementary Fig. 5), phyB might inhibit their expression indirectly through PIF1 and PIF6 transcription factors. In agreement with this, PIF1 activates *RVE1* and *RVE2* transcription (Supplementary Fig. 11a,b).

PIF1 acts as a key transcription factor in modulating far-red/red-light-reversible germination of post-harvest seeds^38^,^39^,^40^. However, PIF1 is not involved in regulating primary dormancy of freshly harvested seeds (Supplementary Fig. 1f)^42^. Conversely, an alternative splice form of PIF6 regulates primary seed dormancy, but not light-mediated seed germination^42^. Here we demonstrate that RVE1 and RVE2 control both the establishment of primary dormancy and red/far-red-light-reversible seed germination. Therefore, seed dormancy and germination are differentially regulated via overlap and distinct pathways by light.

**Figure 5:**
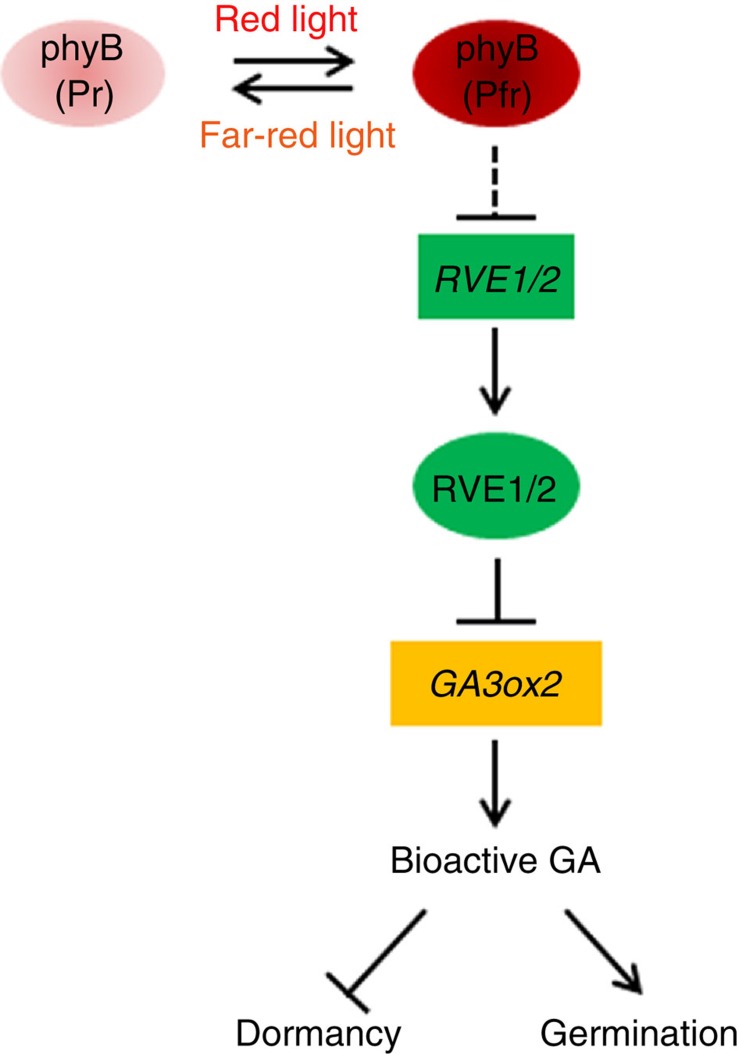
A proposed working model of the phyB-RVE1/2 pathway in regulating seed dormancy and germination. During seed development, the transcripts of *RVE1* and *RVE2* are increasingly accumulated to induce and maintain seeds at the dormant state. Red light triggers the activation of phyB (Pfr form), which represses *RVE1* and *RVE2* expression, leading to the de-suppression of the GA biosynthetic gene *GA3ox2* and the accumulation of bioactive GAs, and consequently the induction of seed germination.

We reveal that, at the molecular level, RVE1 directly binds to the conserved motif of *GA3ox2*, represses its expression and controls bioactive GA accumulation in imbibed seeds (Fig. 4). Other GA biosynthetic genes, such as *GA3ox1*, are indirectly regulated by RVE1. However, ABA level was not affected by RVE1 and RVE2, although the expression of some ABA catabolic genes was regulated by RVE1, suggesting that RVE1/RVE2 specifically control the GA biosynthetic pathway. Consistently, seed responsiveness to GA is mainly controlled by phyB^34^. Therefore, we identify a previously unexplored signalling pathway, consisting of the phyB photoreceptor, RVE1 and RVE2 transcription factors, and GA3ox2, which plays critical roles in regulating GA biosynthesis and eventually primary seed dormancy and light-dependent germination (Fig. 5). As the *rve1/rve2* seeds had ∼80% germination rate (Fig. 1i), other factors and/or signalling mechanisms could be involved in the control of dormancy by light. phyA acts differentially to phyB in promoting seed germination^29^,^31^,^32^. Strikingly, we found that RVE1 also negatively regulated phyA-dependent seed germination (Supplementary Fig. 12). Moreover, RVE1 acts as a transcription factor that regulates different biological functions probably through directly controlling of the expression of downstream genes^46^.

DOG1 is a key factor specific for the induction of primary seed dormancy^3^,^17^. Overexpression of *RVE1* suppresses *dog1* mutant phenotype and that DOG1 acts genetically downstream of phyB (Fig. 2e,f), suggesting that RVE1/RVE2 and DOG1 have similar roles, consistent with their similar expression patterns. Interestingly, RVE1 and DOG1 promote the transcription of each other (Supplementary Fig. 11c,d), although the regulatory mechanism requires further investigation. DOG1 protein levels predict the dormancy status of freshly harvested seeds^17^. It will be of interest to find out whether the proteins of RVE1 and RVE2 act similarly. Previous studies have identified seven QTLs for seed dormancy, three of which (that is, *DOG1*, *DOG4* and *DOG7*) are located on chromosome 5 in *Arabidopsis*^10^. Interestingly, *RVE1* (At5g17300) and *RVE2* (At5g37260) were located approximately within the genomic regions of *DOG4* and *DOG7* loci (Supplementary Fig. 13), pointing to the possibility that *RVE1* and *RVE2* might correspond to *DOG4* and *DOG7*, respectively, and further arguing the importance of RVE1 and RVE2 in controlling seed dormancy. In supporting this proposition, *RVE1* and *RVE2* transcript levels positively correlate with the depth of dormancy in different ecotypes (Fig. 3b,c). Further genetic and molecular experiments are required to verify this possibility in the future.

Light and temperature are two of the important environmental cues affecting seed dormancy and germination. Phytochromes appear to be involved in germination in response to photoperiod and temperature during seed development^54^. Specific phytochrome members contribute to germination differentially, depending on the temperature experienced by seeds during maturation and after dispersal; phyB is important to germination across a wide range of temperature^21^,^55^. It has been known that phytochromes control the expression of GA and ABA metabolic genes^8^,^20^,^32^. A recent study reveals that DOG1 mediates the temperature- and GA-dependent control of germination^56^. Moreover, RVE1 and RVE2 are homologues of the clock oscillator, CCA1, and RVE1 controls daily rhythms of auxin production by integrating the circadian and auxin signalling pathways^44^,^47^. Thus, phytochromes (such as phyB) and RVE1/RVE2 could integrate different environmental inputs (light, circadian and temperature) to modulate endogenous phytohormone metabolic and signalling pathways that control seed dormancy and germination^2^,^3^,^4^, which represents a common genetic adaptation mechanism to various habitats of seeds. Insufficient seed dormancy can lead to pre-harvest sprouting, whereas too much dormancy prevents uniform germination. Thus, optimal level of seed dormancy is an important agronomic trait for crops^57^. The light regulatory networks of seed dormancy and germination might be conserved in species that could help to improve plants' fitness^3^,^58^.

## Methods

### Plant materials and growth conditions

The *rve1-2* (SAIL_326_A01), *rve2-1* (Salk_051843), *phyB-9* (ref. 59), *dog1-2* (ref. 16), *ga1* (Salk_109115) and *pif1-2* (ref. 60) mutants, and *RVE1-OX*^44^, *35S:Myc-RVE1* (ref. 46), *RVE2-OX*^47^, *35S:Myc-PHYB* and *PIF1-OX*^38^ transgenic plants are in the *A. thaliana* Col ecotype. *PHYB-ABO*^61^ is in the Nossen ecotype. Double mutants/transgenic plants were generated by genetic crossing and homozygous lines were used. After sterilization, seeds were sown on 0.6% agar (pH 5.7) plates. GA_3_, DEX and cycloheximide were supplied in the agar medium as indicated in the text. Far-red and red light were supplied by light-emitting diode light sources and white light was supplied by cool white fluorescent lamps. Adult plants were grown in soil with regular irrigation at 22±2 °C, 60–70% humidity and under long-day (16 h light/8 h dark) conditions in a growth chamber, and seeds were harvested at the same time in each batch.

### Dormancy and germination assay

For the seed dormancy assay, seeds were freshly harvested ∼4–5 weeks after fertilization and were surface sterilized and plated on 0.6% agar (pH 5.7) within an hour. Seeds were either stratified or not at 4 °C for 1 to 3 days and the seeds were then incubated in darkness or white light (80 μmol m^−2^ s^−1^) as indicated in the text. Seeds with a protruded radical were considered as germinated seeds. For light-mediated germination assays, harvested seeds were dry-stored in eppendorf tubes in the dark at room temperature for 2 to 5 months, as dry after-ripening is a common method used to relieve dormancy^3^,^7^. For phyB-dependent germination, seeds were exposed to weak white light for 1 h (including sterilization and plating) and incubated in darkness or irradiated with far-red light (3.5 μmol m^−2^ s^−1^) for 5 min to inactivate phyB (phyB-off), or followed by 5 min of red light (20 μmol m^−2^ s^−1^) to activate phyB (phyB-on). For phyA-dependent germination, sterilized and plated seeds were imbibed for 1 h and irradiated with far-red light for 5 min. After 48 h of dark incubation, the seeds were irradiated with far-red light for 12 h. All seeds were then incubated in darkness for 2 days and the germination frequency was determined^39^. At least 100 seeds were used for each genotype in each experiment and three replicates were performed for statistical analysis. All experiments were carried out at least three times with similar results and one representative result is shown.

### Plasmid construction

For the transient expression assay, a 2.5 kb fragment upstream of the *GA3ox2* translational start code was PCR amplified and inserted into the pEASY-Blunt vector (TransGen), generating pEASY-GA3ox2p. The promoter fragment was released from pEASY-GA3ox2p cut with KpnI and PstI, and ligated into the KpnI–PstI site of the pGreenII0800-LUC vector^62^, to generate *GA3ox2p:LUC*. The *RVE1* open reading frame was released from pEASY-RVE1 (ref. 45), digested with EcoRI and SalI, and inserted into the EcoRI–SalI sites of pGAL4BD and pGAL4BD-VP16 (ref. 63) to generate BD-RVE1 and BD-RVE1-VP16, respectively. The RVE1 fragment was also inserted into the MfeI–XhoI sites of pUC18-3HA to produce 35S:RVE1. The RVE1 complementary DNA was re-amplified from pEASY-RVE1 plasmid and digested with NcoI and PmlI. The *RVE1* gene was then inserted into the NcoI–PmlI sites of pCAMBIA1301 (http://www.cambia.org/daisy/cambia/585) to generate pCAMBIA-35S-RVE1. A GR fragment was amplified and digested with SwaI and PmlI, and then ligated into the SwaI–PmlI sites of pCAMBIA-35S-RVE1, generating 35S:RVE1-GR. HIS-RVE1, BD-ERF3RD-VP16, GAL4:LUC and 35S:GUS were produced as described previously^46^,^63^.

To obtain the open reading frame of *GA3ox2* and *PHYB*, first-strand cDNA was reverse transcribed from total RNA extracted from Col wild-type seedlings using oligo(dT)18 primer. The *GA3ox2* and *PHYB* fragments were amplified using high-fidelity *Pfu* DNA polymerase (Invitrogen) and cloned into pEASY, resulting in pEASY-GA3ox2 and pEASY-PHYB, respectively. pEASY-GA3ox2 was cut with EcoRI and SalI to release the *GA3ox2* fragment, which was then ligated into the EcoRI–XhoI site of pRI101-GFP (Takara), giving rise to *35S:GA3ox2*. To facilitate follow-up cloning, two XhoI sites within *phyB* were mutagenized without changing the encoded amino acids, to generate pEASY-phyB-XhoIm. The pEASY-phyB-XhoIm plasmids were digested with MfeI and XhoI, and the released phyB fragment was inserted into the EcoRI–SalI site of pRI101-MYC, resulting in *35S:Myc-PHYB*. To construct *RVE1p:GUS*, a fragment upstream of the translational start code of *RVE1* was amplified by PCR and cloned into pEASY, resulting in pEASY-RVE1p. The pEASY-RVE1p plasmid was digested with EcoRI and SalI to release the *RVE1* promoter, which was then ligated into the EcoRI–SalI sites of the pBI101-GUS vector to generate *RVE1p:GUS*. All amplified fragments were validated by sequencing. Primers used for plasmid construction are listed in Supplementary Table 1.

The binary constructs were electroporated into *Agrobacterium tumefaciens* strain GV3101 and then introduced into the wild type or *rve1-2* mutant via the floral dip method^64^. Transgenic plants were selected on MS plates in the presence of 50 mg l^−1^ kanamycin or hygromycin. Homozygous lines were used in the experiments.

### Gene expression analysis

Sample treatments were described in the respective figure legends. Total RNA was extracted from seeds using Universal Plant Total RNA Extraction Kit (BioTeke) and first-strand cDNA was synthesized using reverse transcriptase (Invitrogen). Quantitative PCR was carried out using the SYBR Premix ExTaq Kit (Takara) following the manufacturer's instructions. Three technical replicates were performed for each sample and the expression levels were normalized to those of *PP2A*. Each experiment was performed at least three times with similar results and one representative result is shown. Primers are listed in Supplementary Table 1.

### ChIP assay

ChIP assay was carried out according to a previous method^65^. Briefly, the *35S:Myc-RVE1*, *35S:Myc-PHYB* or Col seeds were treated under conditions as described in the text and samples were treated with 1% formaldehyde for protein–DNA cross-linking. The chromatin complexes were isolated and sonicated to shear DNA into ∼0.5–2 kb fragments. After spin at 4 °C for 5 min at 13,000 r.p.m., the chromatin supernatant was pre-cleared with salmon sperm-sheared DNA/protein A agarose beads and equally divided into three tubes. The first two tubes were incubated with anti-Myc antibody (1:1,000 dilution, Abcam, ab32) or serum control overnight at 4 °C with gentle agitation. The third tube was used as input control. The samples in first two tubes were incubated with protein A agarose beads for 2 h at 4 °C. The beads were pelleted by centrifugation for 2 min at 13,000 r.p.m. The cross-linked chromatins were eluted and reversed by incubating with 5 M NaCl at 65 °C overnight. The proteins were digested with 14 mg ml^−1^ proteinase K and the DNA fragments were recovered and quantified by quantitative PCR using primers spanning the promoter and coding regions of *GA3ox1*, *GA3ox2*, *RVE1*, *RVE2*, *DOG1* and *UBQ10* control.

### Electrophoresis mobility shift assay

HIS-RVE1 recombinant proteins were expressed in *Escherichia coli* BL21 (DE3) strain and purified using Ni-NTA Agraose (Qiagen). EMSA assay was performed using LightShift Chemiluminescent EMSA Kit (Pierce) according to the manufacturer's protocol. The two complementary oligonucleotides were annealed and labelled with biotin and then incubated with HIS-RVE1 fusion proteins in the absence or presence of excess amounts of unlabelled wild-type or mutant oligonucleotides. The protein–DNA samples were then separated on 5% polyacrylamide gels and signal was captured with a Chemiluminescence Imaging system (Biostep). The oligonucleotides sequences are shown in Supplementary Table 1.

### GUS histochemical analysis

The *RVE1p:GUS* homozygous transgenic seeds were subjected to various light conditions as indicated in the text. The embryo and endosperm were dissected under a stereomicroscope (Olympus) in green light. The samples were incubated in 0.1 M sodium phosphate buffer containing 50 mM K_3_Fe(CN)_6_, 50 mM K_4_Fe(CN)_6_ and 1 mM 5-bromo-4-chloro-3-indolyl-β-D-glucuronide at 37 °C for 3–6 h. GUS staining was examined under a stereomicroscope and images were captured by a digital camera (Olympus).

### GA and ABA content determination

Freshly harvested seeds were imbibed in darkness for 24 h before sampling. For GA determination, seeds were weighted and ground to fine powder in liquid nitrogen. Internal standards of 1 ng g^−1 2^H_2_-GA_1_ and 1 ng g^−1 2^H_2_-GA_4_ were added to the samples followed by extraction with 500 μl solvent (methanol/H_2_O, 80/20, v/v) at 4 °C for 12 h. The supernatants were sequentially passed through the pre-conditioned tandem solid-phase extraction cartridges containing C_18_ adsorbent (50 mg) and strong anion exchange adsorbent (200 mg). The strong anion exchange cartridge was then rinsed with 2 ml of 20% methanol (v/v) and the targeted acidic phytohormones were eluted by 3 ml acetonitrile with 1% formic acid (v/v). The eluent was evaporated under mild liquid nitrogen stream at 35 °C and re-dissolved in 100 μl H_2_O. The solution was acidified with 10 μl formic acid and extracted with 1 ml ether twice. The combined ether phase was dried under nitrogen gas and reconstituted in 100 μl acetonitrile followed by addition of 10 μl triethylamine (20 μmol ml^−1^) and 10 μl 3-bromoactonyltrimethylammonium bromide (20 μmol ml^−1^). The reaction solution was vortexed at 35 °C for 30 min and then evaporated under nitrogen gas. The samples were dissolved in 200 μl 10% acetonitrile (v/v) and subjected for Nano-liquid chromatography–electrospray ionization–quadrupole time-of-flight–mass spectrometry analysis^66^. For ABA measurement, seeds were ground in liquid nitrogen and 45 pmol of ^2^H_2_-ABA internal standard was added to 200 mg of powder. The samples were extracted with 2 ml methanol at −20 °C overnight. After spin at 4 °C for 15 min at 18,000 r.p.m., the supernatant was dried under nitrogen gas and dissolved in 1 ml 5% ammonia solution (v/v). The crude extracts were purified by pre-conditioned Oasis MAX strong anion-exchange column (Waters) and the samples were eluted with 4 ml methanol containing 5% formic acid. The eluent was dried under nitrogen gas and dissolved in 200 μl 80% methanol (v/v) and subjected for ultra-performance liquid chromatography tandem mass spectrometry analysis^67^.

### LUC transient expression assay

For transient expression of *GA3ox2* by RVE1, the reporter plasmid GA3ox2p:LUC and 35S:RVE1 effector or vector control were co-transformed into *Arabidopsis* protoplasts. The protoplasts were pelleted and resuspended in 100 μl of × 1 cell culture lysis reagent (Promega). Luminescence activities of firefly and *Renilla* were measured using Dual-Luciferase Reporter Assay System reagent in a Modulus Luminometer/Fluorometer equipped with a luminescence kit (Promega). Five microlitres of the extract was first mixed with 15 μl of LAR II reagent to determine firefly luminescence (LUC_firefly_). Fifteen microlitres of Stop and Glo assay reagent was then added to measure the *Renilla* luminescence (LUC_Renilla_). The relative reporter expression level was expressed as the LUC_firefly_/LUC_Renilla_ ratio. For RVE1 transcriptional activation activity assay, the reporter plasmid GAL4p:LUC, effector constructs (DB-RVE1, DB-VP16, DB-RVE1-VP16, DB-ERF3RD-VP16 or empty vector) and 35S:GUS internal control were co-transformed into *Arabidopsis* protoplasts. After cell lysis, 5 μl of the extract was mixed with 15 μl of LUC Assay Substrate (Promega) to determine LUC activity. For GUS enzymatic assay, 5 μl of the extract was incubated with 45 μl 4-methylumbelliferyl β-D-glucuronide assay buffer (50 mM sodium phosphate pH 7.0, 1 mM 4-methylumbelliferyl β-D-glucuronide, 10 mM EDTA, 10 mM β-mercaptoethanol, 0.1% sarkosyl and 0.1% Triton X-100) at 37 °C for 15 min and the reaction was stopped by adding 950 μl of 0.2 M Na_2_CO_3_. GUS fluorescence was measured using an ultraviolet fluorescence optical kit. The relative reporter expression level was expressed as the LUC/GUS ratios.

### Data availability

The authors declare that all data supporting the findings of this study are available in the manuscript and its Supplementary Information files or are available from the corresponding author upon request.

## Additional information

**How to cite this article:** Jiang, Z. *et al.* Phytochrome B and REVEILLE1/2-mediated signalling controls seed dormancy and germination in *Arabidopsis*. *Nat. Commun.* 7:12377 doi: 10.1038/ncomms12377 (2016).

## Supplementary Material

Supplementary Information Supplementary Figures 1 - 13, Supplementary Table 1 and Supplementary References
