# Supplementary material for: Phytochrome B and REVEILLE1/2-mediated signalling controls seed dormancy and germination in Arabidopsis
Source: Nat Commun. 2016 Aug 10;7:12377. doi: 10.1038/ncomms12377 (PMC4987513; doi:10.1038/ncomms12377)
Supplement: Supplementary Information — Supplementary Figures 1 - 13, Supplementary Table 1 and Supplementary References [file ncomms12377-s1.pdf]

Supplementary Information

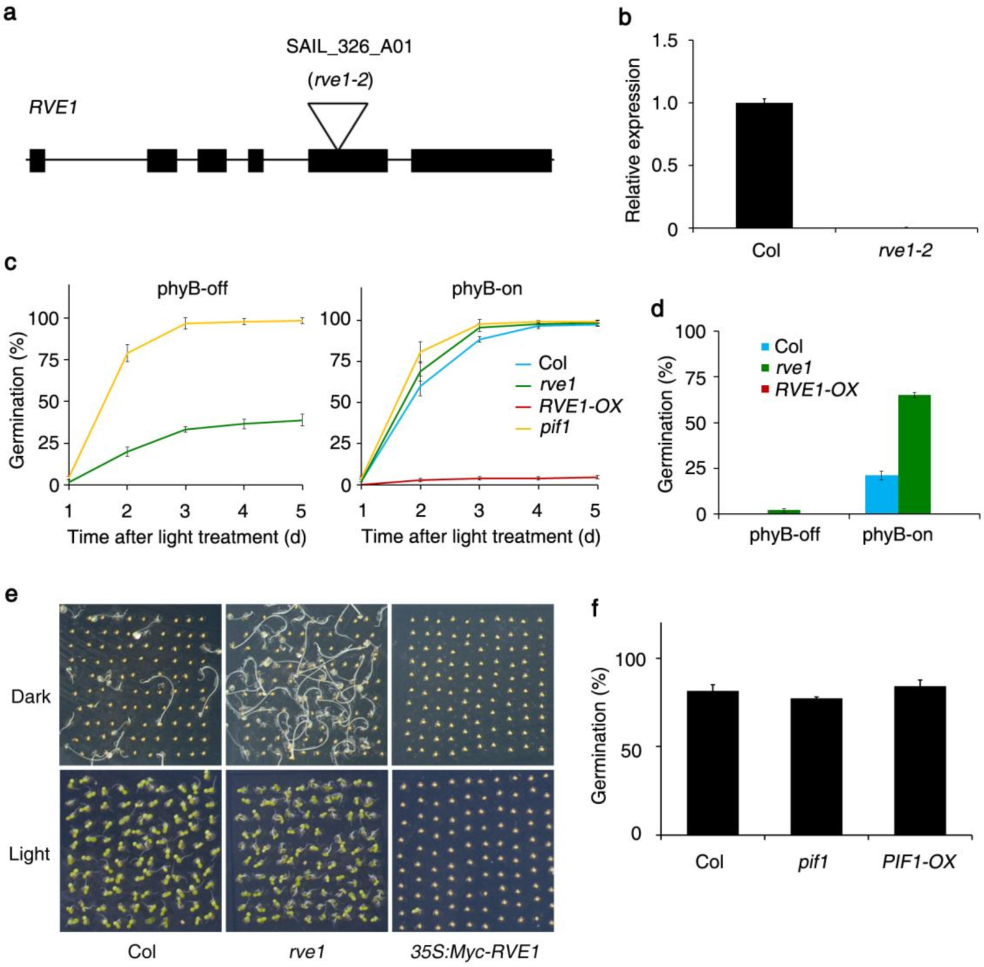

Supplementary Fig. 1. Seed dormancy and germination responses of *RVE1* and *PIF1*.

(a) Diagram of *RVE1* and the T-DNA insertion of the *rve1-2* mutant (SAIL_326_A01). Black boxes represent exons and lines between the boxes indicate introns. Triangles denote T-DNA insertions. (b) *RVE1* transcript level in Col and the *rve1-2* mutant, as determined by RT-PCR. (c) Time course study of seed germination under phyB-off and phyB-on conditions. Post-harvested seeds were treated as in Fig.1a and germination frequencies were recorded every 1 d after light treatment. (d) Seed dormancy response under phyB-off and phyB-on conditions. Freshly harvested seeds were treated as in Fig. 1a and germination rate was determined after 5 d. (e) Dormancy phenotype of freshly harvested *35S:Myc-RVE1* seeds grown in darkness or under white light for 5 d. (f) Dormancy phenotype of freshly harvested seeds of the *pif1* mutant and *PIF1* overexpression line under light for 4 d. For (b-d, f), mean ± SD, n=3.

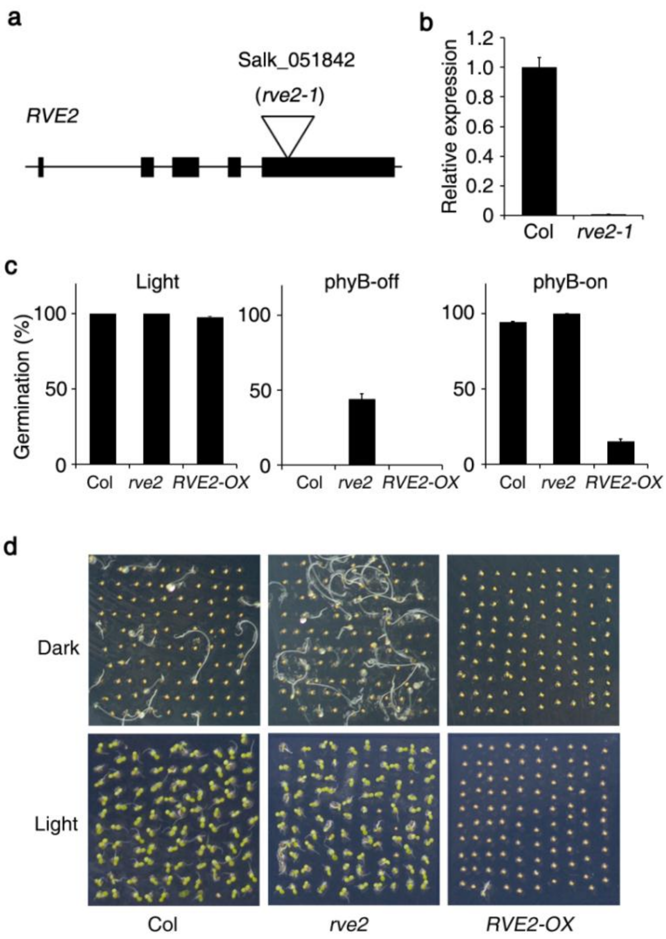

Supplementary Fig. 2. Phenotype of the *rve2* mutant and *RVE2* overexpression line. (a) Diagram of *RVE2* and the T-DNA insertion in the *rve2-1* mutant (Salk_051842). Black boxes represent exons and lines between the boxes indicate introns. Triangles denote T-DNA insertions. (b) *RVE2* transcript level in Col and the *rve2-1* mutant, as determined by RT-PCR. (c) Seed germination frequency of Col, *rve2-1*, and *RVE2-OX* under white light, phyB-off, and phyB-on conditions. (d) Germination percentage of freshly harvested seeds of Col, *rve2-1,* and *RVE2-OX* grown in darkness or under white light. It should be noted that the Col controls in (d) are same as those in Supplementary Fig. 1e, since the two experiments were done at the same time. For b-c, mean ± SD, n=3.

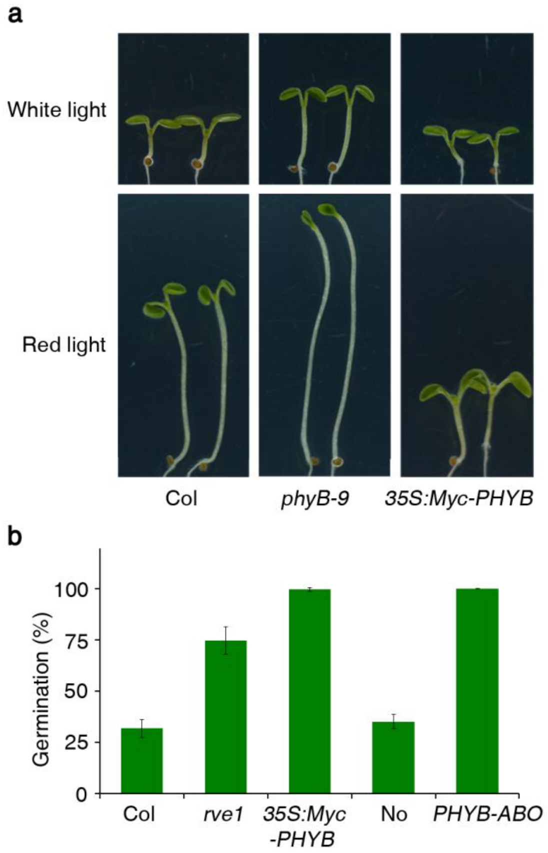

Supplementary Fig. 3. Characterization of *35S:Myc-PHYB*.
(a) Col, *35S:Myc-PHYB* transgenic and *phyB-9* mutant seedlings were grown in white light or red light for 5 d. Overexpression of *PHYB* leads to short hypocotyls, whereas *phyB* mutants display long hypocotyls. (b) Dormancy response of *PHYB* overexpression seeds. Freshly harvested dry seeds were stored for 1 month and were sown on wet filter paper and immediately imbibed in darkness and the phenotype was observed after 3 d of dark incubation. Mean ± SD, n=3.

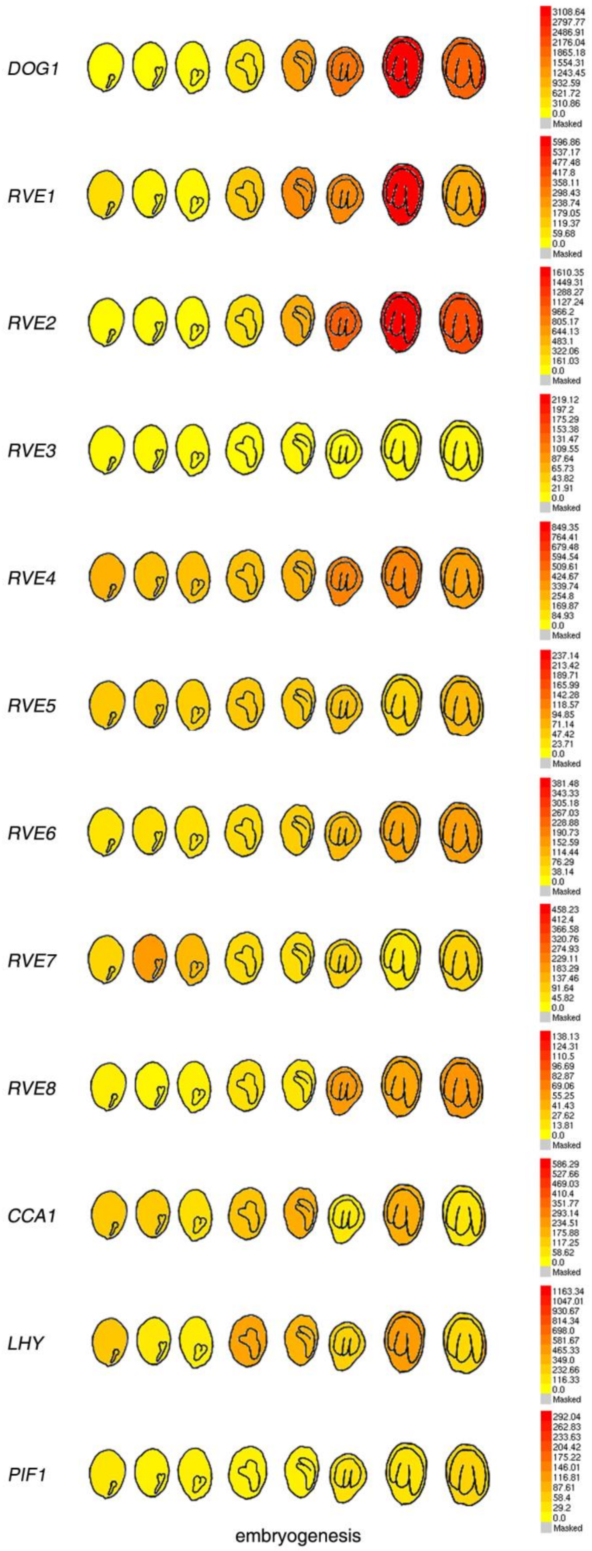

Supplementary Fig. 4. Expression mode of various genes during seed development. Data were collected from http://bar.utoronto.ca/efp/cgi-bin/efpWeb.cgi (ref. 1). The expression of *DOG1*, *RVE1*, and *RVE2* starts to increase during the torpedo stage and peaks at the curly cotyledon stage.

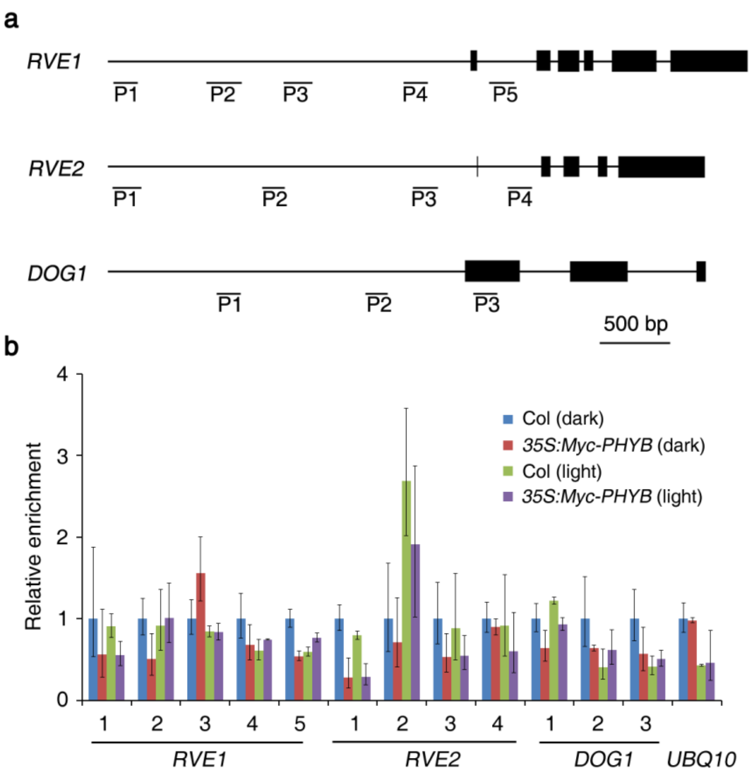

Supplementary Fig. 5. phyB does not associate with genomic regions of *RVE1*, *RVE2* and *DOG1*.

(a) Diagrams of *RVE1*, *RVE2* and *DOG1* genes. Black boxes indicate exons and P1 to P5 denote regions for PCR. (b) ChIP assay showing relative enrichment of *RVE1*, *RVE2*, and *DOG1* DNA precipitated in *35S:Myc-PHYB* or Col by Myc antibody. Freshly harvested *35S:Myc-PHYB* and Col seeds were incubated in the dark or under light condition for 24 h. Mean ± SD, n=3.

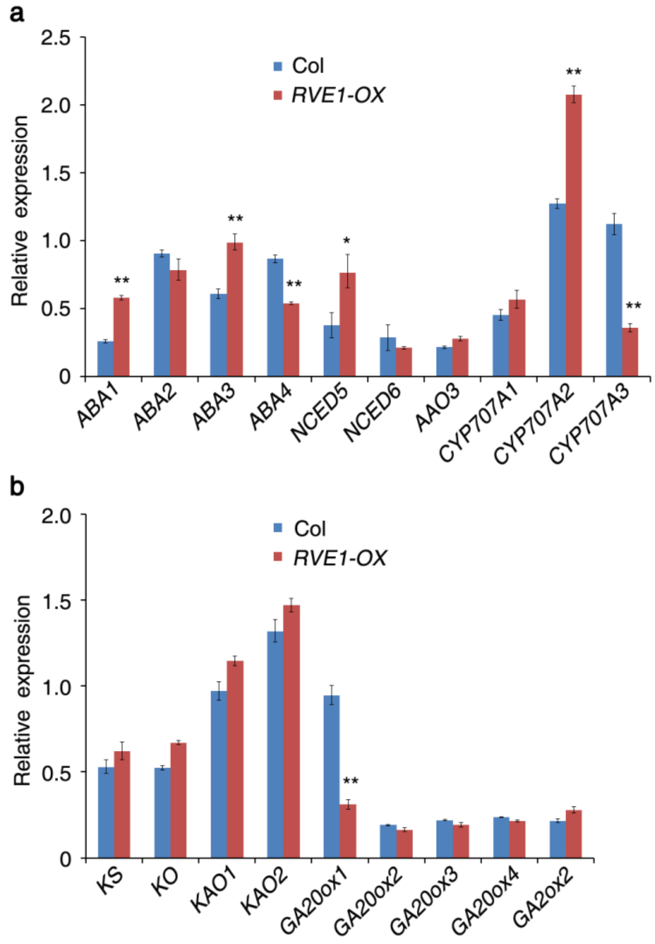

Supplementary Fig. 6. Relative expression level of ABA and GA metabolic genes. (a) Expression of ABA metabolic genes. (b) Expression of GA metabolic genes. Post-harvest Col and *RVE1-OX* seeds were incubated under phyB-on conditions. The transcripts of other ABA metabolic genes, including *NCED2*, *NCED3* and *CYP707A4*, and GA metabolic genes, including *GA2ox1*, *GA2ox3* to *GA2ox8*, *GA20ox5*, *GA3ox3* and *GA3ox4*, were not detectable, likely due to very low expression. Asterisks denote statistically significant difference (**P<0.01; *P<0.05, Student's *t* test). Mean ±SD, n=3.

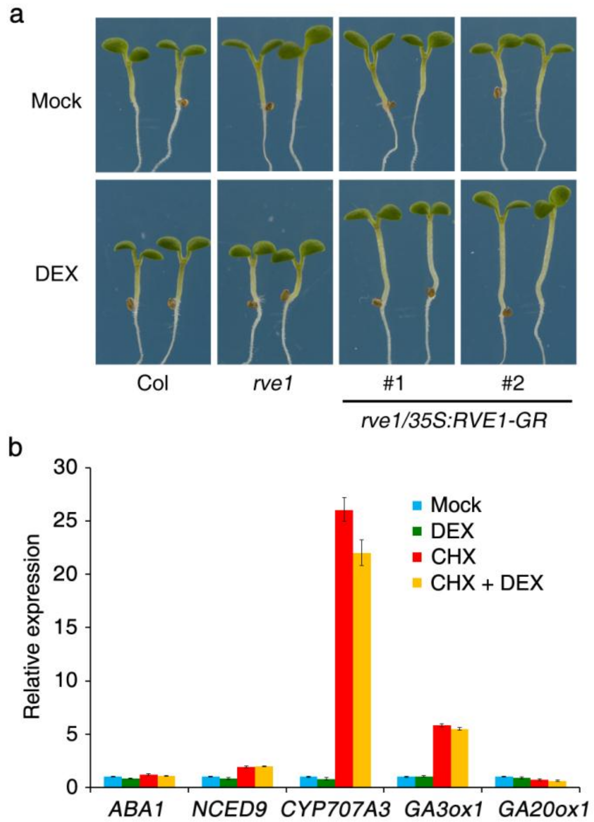

Supplementary Fig. 7. Gene expression in the *35S:RVE1-GR* transgenic seeds.

(a) Seedling phenotype of *35S:RVE1-GR* without or with 5 μM DEX treatment. (b) Relative expression of various ABA or GA metabolic genes in *35S:RVE1-GR* seeds without (Mock) or with 5 μM DEX and/or 50 μM CHX treatments. Mean ± SD, n=3.

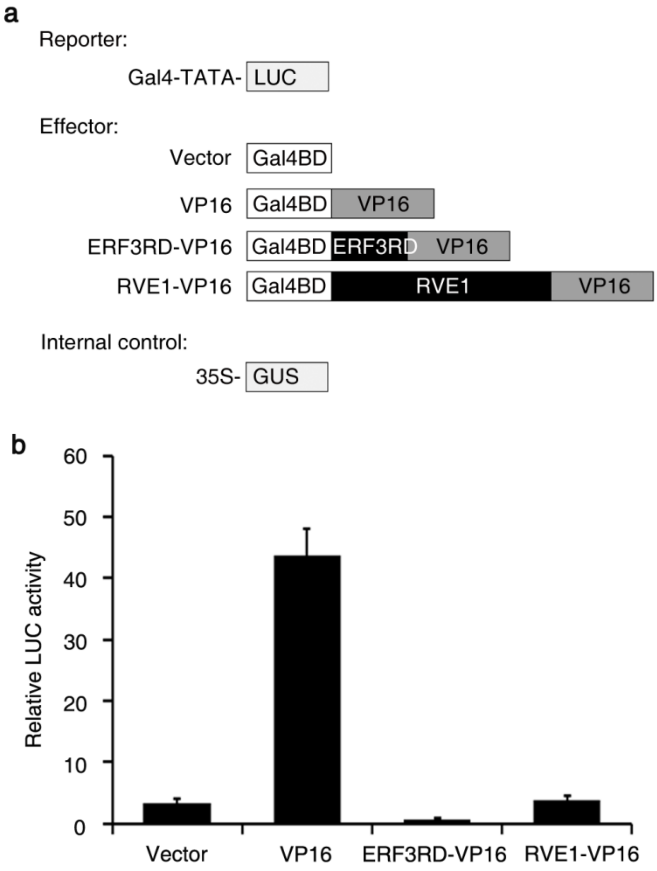

Supplementary Fig. 8. RVE1 possesses transcriptional repression activity.
(a) Diagram of various constructs. (b) Relative LUC/GUS activity. The repression domain of ERF3 was used as a control. Mean ± SD, n=4.

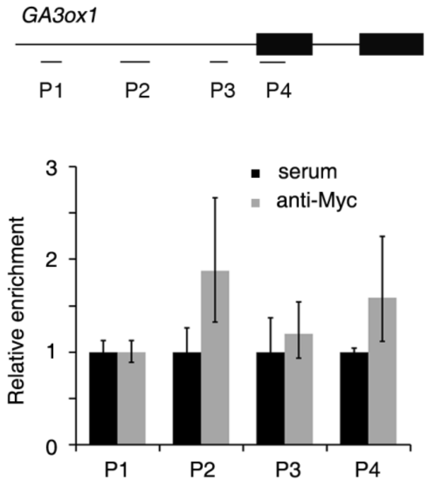

Supplementary Fig. 9. ChIP assay of *GA3ox1*. The experiment was performed as shown in Fig. 4d. The top diagram indicates the genomic structure of *GA3ox1* and PCR fragments (P1 to P4) in the ChIP assay. Mean ± SD, n=3.

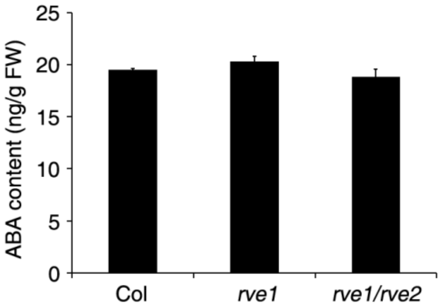

Supplementary Fig. 10. ABA contents. Freshly harvested Col, *rve1*, and *rve1/rve2* seeds were imbibed in darkness for 24 h. Mean ± SD, n=3.

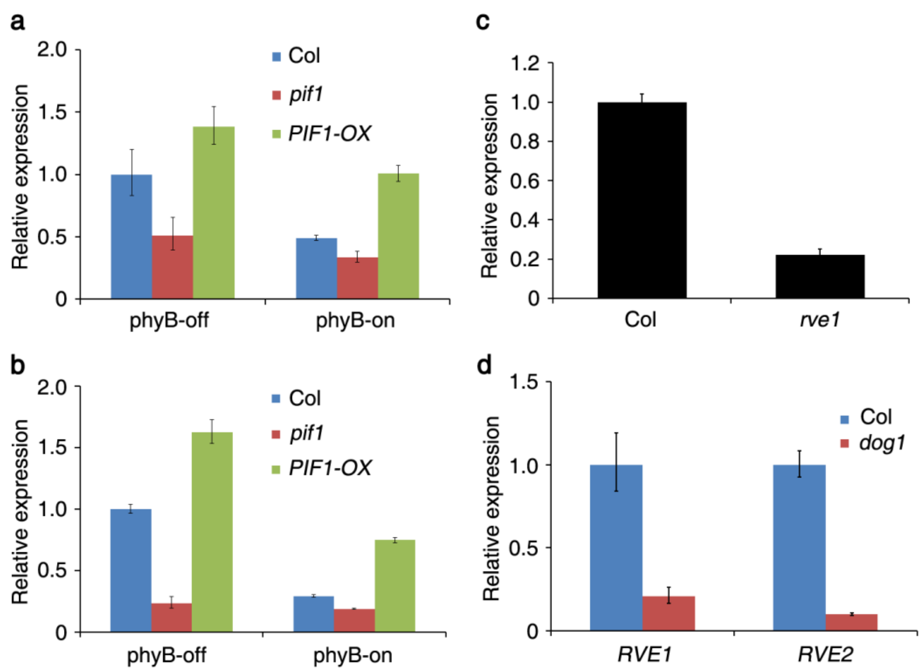

Supplementary Fig. 11. Transcriptional regulation among *RVE1*, *DOG1*, and *PIF1*. (a and b) Relative expression levels of *RVE1* (a) and *RVE2* (b) in the post-harvested seeds of *pif1* mutant and *PIF1* overexpression line grown under phyB-off and phyB-on conditions. (c) Relative *DOG1* expression in the freshly harvested Col and *rve1* seeds imbibed in darkness for 12 h. (d) Relative expression levels of *RVE1* and *RVE2* in the post-harvested seeds of Col and *dog1* mutant grown under phyB-off condition. Mean ± SD, n=3.

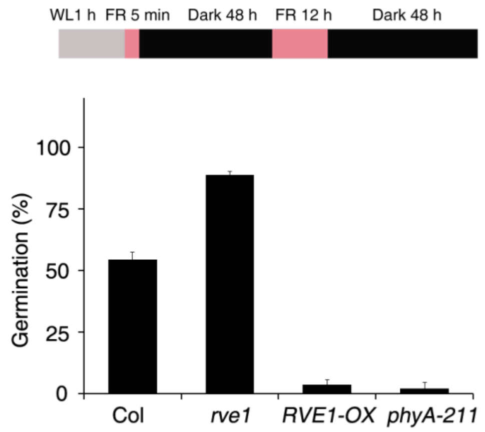

Supplementary Fig. 12. RVE1 regulates phyA-mediated seed germination.
Post-harvested seeds were imbibed for 1 h and irradiated with far-red light for 5 min. After 48 h of dark incubation, the seeds were irradiated with far-red light for 12 h, and germination frequency was determined after 48 h in darkness. *phyA* mutant is set as a control (ref. 2). WL, white light; FR, far-red light.

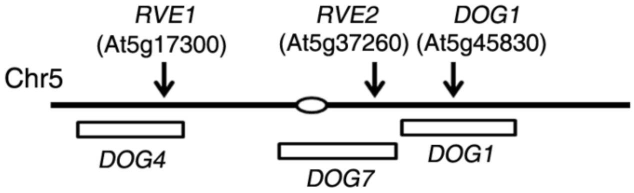

Supplementary Fig. 13. Diagram of the location of three dormancy QTLs (ref. 3) and *RVE1*, *RVE2*, and *DOG1* genes on chromosome 5. Not to scale.

Supplementary Table 1. List of primers used in this study.

Gene AGI code	Oligo name	Sequence (5'-3')	Purpose
RVE1 AT5G17300	RVE1p- F	CGTAGACTTACGTTATGGATAGACCC	Construction of RVE1p:GUS
	RVE1p-SalI-R	GTCGACAACTTCCCGGATTAAGATCTCG	
	RVE1-EcoRI-F	GAATTCATGGCGTCGTCTCCGTTGACTG	Construction of BD-RVE1, BD-RVE1-VP16, 35S:RVE1
	RVE1-SalI-R	GTCGACTAAGTGGAGATGAATCTCATGC	
	RVE1-NcoI-F	GCGCCATGGACATGGCGTCGTCTCCGTTGAC	Construction of 35S:RVE1-GR
	RVE1-PmlI/SwaI-R	GCGCACGTGATTTAAATCTAAGTGGAGATGAATCTCATG	
	GR-SwaI-F	GCGATTTAAATAAAGGGATTCAGCAAGCC	
	GR-PmlI-R	GCGCACGTGTCATTTTTGATGAAACAG	
	RVE1-QF	CTCCTCGTCCCAAGAGAAAG	qRT-PCR
	RVE1-QR	GTGGACAACACAGAGGTTGG	
	rve1-LP	AACCAGTGTTTGATCCAGTCG	Genotyping of *rve1-2*
	rve1-RP	CAAAGACCGCAGTTCAGATTC	
	RVE1-CHIP-1F	AGTAGACAACAGCCACACAC	ChIP-qPCR
	RVE1-CHIP-1R	AATGACGAGAAGGAGGGTTC	
	RVE1-CHIP-2F	GATTGTGATTCAAAACCATTTC	
	RVE1-CHIP-2R	AATGAGATCCACAAAATGGG	
	RVE1-CHIP-3F	TTGATTAAGATATCCACTTCGG	
	RVE1-CHIP-3R	ATAGGATTTGTTTGCGGAAC	
	RVE1-CHIP-4F	ATGAGAAAGAGCGACACGTG	
	RVE1-CHIP-4R	ACAAAAAGGAGGGTGATGCC	
	RVE1-CHIP-5F	GCAAGTTCGTTGCAATTCAT	
	RVE1-CHIP-5R	GAACATTCATTCACAAAGTCCAA	
RVE2 AT5G37260	RVE2-QF	CTAACCGGATCCAAGCTGAT	qRT-PCR
	RVE2-QR	GAACCTAATCCATCTGAGCCA	
	rve2-LP	CAAGGATCTCAATTTCTAACTGG	Genotyping of *rve2-1*
	rve2-RP	TGACTTTTGTTGGTTCTTCTATGG	
	RVE2-CHIP-1F	TGAAGTTGACGGCTACTGAA	ChIP-qPCR
	RVE2-CHIP-1R	CCTAAATGAGGGCCAAGAAC	
	RVE2-CHIP-2F	ATACTCAGAAAGGTATCAAGGG	
	RVE2-CHIP-2R	AATCTCAACTAGACGGTGTGT	
	RVE2-CHIP-3F	TTAAACAAGACACGCGCTCA	
	RVE2-CHIP-3R	GTGGAGGATTTCTTCTTCGCT	
	RVE2-CHIP-4F	TTCGATTGTGGCAGGTTAAA	
	RVE2-CHIP-4R	CGCAACTAGAAGAGGTAGAC	
GA3ox2 AT1G80340	GA3OX2-EcoRI-F	GAATTCATGAGTTCAACGTTGAGCGA	Construction of 35S:GA3ox2
	GA3OX2-SalI-R	GTCGACATTTCTAATAATGGAAAGAGAT	
	GA3OX2p-KnpI-F	GGTACCATCACATCTACGACATTTCCCTC	Construction of

	GA3OX2p-PstI-R	CTGCAGGCTAAAAGGCTTATGTGTTTATATTTTGGC	GA3ox2p:LUC
	GA3OX2-QF	AAGGTTTCACCGTTATTGGC	qRT-PCR
	GA3OX2-QR	ACCTAATGCGAACCACATCA	
	GA3OX2-CHIP-1F	TTGTAACGGTATAAGGCTTGGC	ChIP-qPCR
	GA3OX2-CHIP-1R	GCCTCTCACTTGCTAGTGTTATA	
	GA3OX2-CHIP-2F	TTGTTTTAAGCTGTCTATTTCCAAG	
	GA3OX2-CHIP-2R	CTTTTGGTGGAGAAGAGGAGTG	
	GA3OX2-CHIP-3F	GCATCCCATTCACATCCCACTCTC	
	GA3OX2-CHIP-3R	TGGTGATCTGGAACGCTCCCC	
	GA3OX2-CHIP-4F	CCTTTGGCTACATGACGATTTCTA	
	GA3OX2-CHIP-4R	GTCATGAGGGTCGAGTCTGT	
	GA3OX2-EMSA-3F	GTTTAACGATATTAGTTTTTGTTTAACGATATTAGTTTT TGTTTAACGATATTAGTTTTT	EMSA
	GA3OX2-EMSA-3R	AAAAACTAATATCGTTAAACAAAAACTAATATCGTTAA ACAAAAACTAATATCGTTAAAC	
	GA3OX2-EMSA-3Fm	GTTTAACGGGATAGGTTTTTGTTTAACGGGATAGGTTT TTGTTTAACGGGATAGGTTTTT	
	GA3OX2-EMSA-3Rm	AAAAACCTATCCCGTTAAACAAAAACCTATCCCGTTAA ACAAAAACCTATCCCGTTAAAC	
	GA3OX2-EMSA-4F	AAGTATCATATCATACCAAAAAGTATCATATCATACCA AAAAGTATCATATCATACCAAA	
	GA3OX2-EMSA-4R	TTTGGTATGATATGATACTTTTTGGTATGATATGATACT TTTTGGTATGATATGATACTT	
	GA3OX2-EMSA-4Fm	AAGTACGTCTCAGTACCAAAAAGTACGTCTCAGTACCA AAAAGTACGTCTCAGTACCAAA	
	GA3OX2-EMSA-4Rm	TTTGGTACTGAGACGTACTTTTTGGTACTGAGACGTAC TTTTTGGTACTGAGACGTACTT	
GA3ox1 AT1G15550	GA3OX1-QF	AAATGTGGTCCGAAGGTTTC	qRT-PCR
	GA3OX1-QR	CATCAATTTCGATGCCAACT	
	GA3OX1-CHIP-1F	GACGATAACGTTTTCTTCCACT	ChIP-qPCR
	GA3OX1-CHIP-1R	TATGCTGCAAAGTAGACGATTG	
	GA3OX1-CHIP-2F	CTTTATTCTATACGCCTCTTGC	
	GA3OX1-CHIP-2R	AAAATACTTTGGAAGGGAACG	
	GA3OX1-CHIP-3F	GGATAGATACGGTTTAACTTC	
	GA3OX1-CHIP-3R	AAAGCACTTGTTTTGGTCCAA	
	GA3OX1-CHIP-4F	TTAGAGGCCATCCCATTCA	
	GA3OX1-CHIP-4R	CTCAATGTCTTGGAGAAGTC	
UBQ10 AT4G05320	UBQ10-CHIP-F	TCCAGGACAAGGAGGTATTCCTCCG	ChIP-qPCR
	UBQ10-CHIP-R	CCACCAAAGTTTTACATGAAACGAA	
DOG1 AT5G45830	DOG1-QF	AAGAAGACGCAGCGGATATT	qRT-PCR
	DOG1-QR	TTGTCGAGAGCTTGATCCAC	
	DOG1-F	ATGGGATCTTCATCAAAGAACATCGAAC	Genotyping of *dog1-2*
	dog1-2-R2	GAATTTTGGTATAGATCTATGGTTCGGAATC	

	DOG1-CHIP-1F	TAACGACTAACGACAACCGT	ChIP-qPCR
	DOG1-CHIP-1R	ACAAGCGGTCCACAATATCT	
	DOG1-CHIP-2F	ATTAGTTTGTGAGTGTGTCGG	
	DOG1-CHIP-2R	ACTAATAGAGAGTGCGAGTTGT	
	DOG1-CHIP-3F	CTGATCTTGCTCACCGATGT	
	DOG1-CHIP-3R	TGTTGCGGAGAAACTGAGTC	
PHYB AT2G18790	phyB-F	GGTACCCAATTGATGGTTTCCGGAGTCGGGGGTA	Construction of 35S:Myc-PHYB
	phyB-R	CTCGAGATATGGCATCATCAGCATCATGT	
CPS AT4G02780	ga1-LP	CAGACCCGAGACAGTAACTGC	Genotyping of *ga1*
	ga1-RP	TCTCTACTCGAGGCAAGCTTG	
KS AT1G79460	KS(GA2)-QF	ACTCAATGGTTCTGCTGTGG	qRT-PCR
	KS(GA2)-QR	CTTCACGGTGTATGGACTGG	
KO AT5G25900	KO-QF	TTTCGGTGTAGCATTGAAGC	
	KO-QR	CAACATCAATTGCACCTTCC	
KAO1 AT1G05160	KAO1-QF	TCATCTGCGTAAGCTCACCT	
	KAO1-QR	TCCAGGAATATTGACTGCCA	
KAO2 AT2G32440	KAO2-QF	CCTTCAAGATGGGAGGGATA	
	KAO2-QR	CTTTCCACCCGGTATTTGAG	
GA20ox1 AT4G25420	GA20OX1-QF	TTTCATGGCTCTATCGAACG	
	GA20OX1-QR	TTCGGACACAAGAAGAATGC	
GA20ox2 AT5G51810	GA20OX2-QF	GTCGACAATCAATGGCAATC	
	GA20OX2-QR	TCTCGCGCTCTCTCTATTCA	
GA20ox3 AT5G07200	GA20OX3-QF	GTGGTGAACATAGGCGACAC	
	GA20OX3-QR	CCCTTTCGGACATAGGAAGA	
GA20ox4 AT1G60980	GA20OX4-QF	CATTGGCGACACTTTAATGG	
	GA20OX4-QR	CACCACTTTGTCCACTTTCG	
GA2ox2 AT1G30040	GA2OX2-QF	GAAGAGTGACTCGTGCCTGA	
	GA2OX2-QR	GACCCGCCGTGTTATTAGAT	
ABA1 AT5G67030	ABA1-qRT-F	GACTGGGTCCTTGGAGGTAA	
	ABA1-qRT-R	CATCGGCTTTGTCAGTGAGT	
ABA2 AT1G52340	ABA2-qRT-F	TCCAAGCATGCTGTTCTAGG	
	ABA2-qRT-R	AAATGAGCCAAAGCGAGTTT	
ABA3 AT1G16540	ABA3-qRT-F	GTGATACGTTGGCCACTTTG	
	ABA3-qRT-R	GACCCTGAACCATCCATTCT	
ABA4 AT1G67080	ABA4-qRT-F	ACTCTTGCTTCTGCTTGGAT	
	ABA4-qRT-R	ATTCCAACCGGACAGAAGAG	
NCED5 AT1G30100	NCED5-QF	TCCGGTAACGAAGGAGCTAT	
	NCED5-QR	GTCCGGTGATTTCTCACCTT	
NCED6 AT3G24220	NCED6-QF	ACCGGATTGTTTCTGTTTCC	
	NCED6-QR	ACGACGATAACTGGGTCTCC	
NCED9 AT1G78390	NCED9-QF	CCGGTTATCTCGGAACAAGT	
	NCED9-QR	CCAGATAAGCATACCGGGTT	
AAO3	AAO3-QF	GAAGGTCTTGGAAACACGAAGAA	

AT2G27150	AAO3-QR	GAAATACACATCCCTGGTGTACAAAAC	
CYP707A1	CYP707A1-qRT-F	TGTCCTGGAAATGAATTAGCC	
AT4G19230	CYP707A1-qRT-R	GAATGGCCCATACTGAATCC	
CYP707A2	CYP707A2-QF	ATCCTCCTTCACCACCTCAC	
AT2G29090	CYP707A2-QR	CCTTCTTGGGTACAGGGAAA	
CYP707A3	CYP707A3-QF	TGTCCAGGCAATGAGTTAG	
AT5G45340	CYP707A3-QR	GGCAATAGGCAATCCATTCT	
PP2A	PP2A-QF	TATCGGATGACGATTCTTCGTGCAG	
AT1G13320	PP2A-QR	GCTTGGTCGACTATCGGAATGAGAG	

Supplementary References

1. Winter, D., Vinegar, B., Nahal, H., Ammar, R., Wilson, G.V. & Provart, N.J. An "Electronic Fluorescent Pictograph" browser for exploring and analyzing large-scale biological data sets. *PLoS ONE* **2**, e718 (2007).
2. Oh, E., Kim, J., Park, E., Kim, J-I., Kang, C. & Choi, G. PIL5, a phytochrome-interacting basic helix-loop-helix protein, is a key negative regulator of seed germination in *Arabidopsis thaliana*. *Plant Cell* **16**, 3045-3058 (2004).
3. Alonso-Blanco, C., Bentsink, L., Hanhart, C.J., Blankestijn-De Vries, H. & Koornneef, M. Analysis of natural allelic variation at seed dormancy loci of *Arabidopsis thaliana*. *Genetics* **164**, 711–729 (2003).
